# Maternal Cardiac Arrest: A Practical and Comprehensive Review

**DOI:** 10.1155/2013/274814

**Published:** 2013-07-17

**Authors:** Farida M. Jeejeebhoy, Laurie J. Morrison

**Affiliations:** ^1^Division of Cardiology, William Osler Health System and Division of Cardiology, Department of Medicine, Faculty of Medicine, University of Toronto, Toronto, ON, Canada L6W 3W8; ^2^Rescu, Keenan Research Centre, Li Ka Shing Knowledge Institute, St. Michael's Hospital and Division of Emergency Medicine, Department of Medicine, Faculty of Medicine, University of Toronto, Toronto, ON, Canada M5B 1W8

## Abstract

Cardiac arrest during pregnancy is a dedicated chapter in the American Heart Association Guidelines for Cardiopulmonary Resuscitation and Emergency Cardiovascular Care; however, a robust maternal cardiac arrest knowledge translation strategy and emergency response plan is not usually the focus of institutional emergency preparedness programs. Although maternal cardiac arrest is rare, the emergency department is a high-risk area for receiving pregnant women in either prearrest or full cardiac arrest. It is imperative that institutions review and update emergency response plans for a maternal arrest. This review highlights the most recent science, guidelines, and recommended implementation strategies related to a maternal arrest. The aim of this paper is to increase the understanding of the important physiological differences of, and management strategies for, a maternal cardiac arrest, as well as provide institutions with the most up-to-date literature on which they can build emergency preparedness programs for a maternal arrest.

## 1. Introduction

Managing a maternal cardiac arrest is an extremely challenging and trying task for emergency department (ED) staff as there are two patients, the mother and the fetus. Since out-of-hospital maternal cardiac arrests carry the worst outcomes, emergency medical services (EMS) also play an integral part in the management process [1]. The optimal management of a maternal cardiac arrest requires the participation of several different nonemergency teams, as well as the use of specialized equipment [2, 3], neither of which are part of the usual emergency department code protocols. This would include the obstetrical team, the anesthesia team, and the neonatal team, as well as equipment for a perimortem cesarean section and neonatal resuscitation. Ensuring that the right staff and equipment arrive at the code scene in a timely manner is imperative but often practically difficult. 

 Education and training are essential to managing a maternal cardiac arrest; however, the current skill, knowledge, and implementation of existing guidelines among staff are poor [4–6]. Current ACLS training courses do not routinely include a comprehensive review of maternal resuscitation, and while specialized courses are being developed, they are not yet widely available [7]. 

Cardiac disease is the number one cause of maternal mortality based on a United Kingdom database that holds the largest population-based data on this specific group [8]. Women are deferring pregnancy to older ages, and more women with complex health problems are choosing to go through pregnancy [8, 9]. Moreover, even if these patients are being followed in high-risk centres, their residence may be located closer to smaller hospital centres. Thus, care for these patients when they are critically ill may occur in any emergency department. Critical events can occur at any time in the obstetric population and emergency medical services could be the first point of contact during such events. This means that patients could potentially arrive at any emergency department and makes excellent communication between EMS providers and the receiving emergency department crucial. 

 Pregnant women are usually directed to the labour and delivery floor when they become ill during the later stages of pregnancy. Implementation papers directly affecting obstetrical staff and in-hospital cardiac arrest teams have been published to encourage awareness and adaptation of existing protocols to be more in line with the science and current evidence guidelines [2]. Having a proactive approach to emergency preparedness for the pregnant patient in cardiopulmonary arrest is essential for all emergency departments. Understanding the physiological changes of pregnancy, the direct and indirect evidence on maternal cardiac arrest resuscitation approaches and how both have contributed to our current resuscitation guidelines are important first steps to adapting local resuscitation protocols for maternal cardiac arrests.

## 2. Current Science and Guidelines

### 2.1. Systematic Review on Maternal Resuscitation

Recently, the first systematic review on the management of cardiac arrest during pregnancy was published and highlighted the lack of science in the area of maternal resuscitation [10]. The five studies in the area of maternal cardiac arrest reported several important findings. The first finding was that the transthoracic impedance does not change with pregnancy, and, therefore, current defibrillation energy recommendations are the same in both the pregnant and nonpregnant patient [10, 11]. Second, although chest compressions are feasible in the tilted position (nonphysiological data) [12], the maximum possible resuscitative force with chest compressions declines as the angle of inclination increases [10, 13]. The remaining two studies looked at perimortem cesarean sections (PMCSs). One retrospective cohort from The Netherlands reported that PMCS was mainly considered for fetal viability; however, after the completion of a training course on the importance of PMCS for maternal benefit, the frequency of performed PMCS significantly increased [10, 14]. While this retrospective cohort study found poor outcomes with PMCS, it was noted that PMCSs were not performed within the recommended 4-5 minute timeframe and many unnecessary, time-consuming delays were made [10, 14]. Finally, the last study was a case series on PMCS [10, 15] which reported that very few PMCS (8/38) were performed within the recommended 4-5-minute timeframe after onset of maternal cardiac arrest. Yet, despite these time delays for PMCS, positive neonatal and maternal outcomes were still possible [15]. Several women had a sudden and dramatic improvement in their hemodynamics, with a return of pulse and blood pressure immediately after PMCS. The neonates that had higher survival outcomes tended to be older in their gestational age at birth [15]. 

### 2.2. American Heart Association 2010 Guidelines

**Figure 1:**
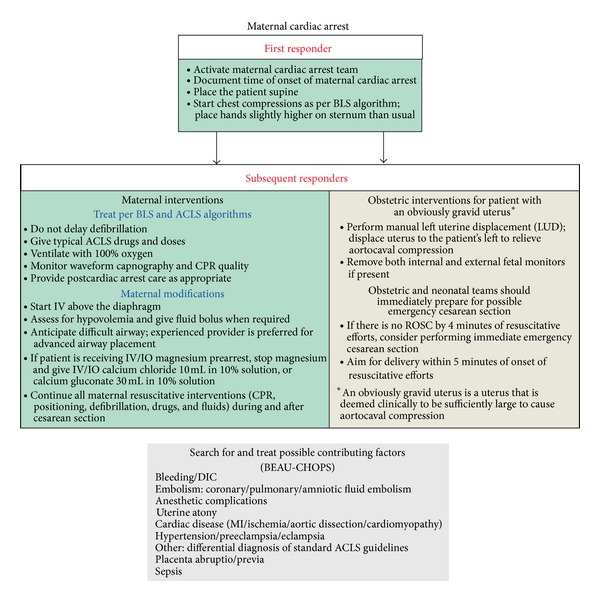
Maternal Cardiac Arrest Algorithm [3].

The International Liaison Committee on Resuscitation (ILCOR) published the most recent science on maternal resuscitation in 2010 [16–18]. This consensus on science and treatment recommendations, combined with expert opinions and agreement, led to the development of the most recent American Heart Association (AHA) Guidelines for Cardiopulmonary Resuscitation and Emergency Cardiovascular Care with a chapter specifically dedicated to maternal resuscitation [3]. These guidelines published the first evidence-based algorithm for the management of cardiac arrest during pregnancy (Figure 1) [3]. This algorithm should be the basis for emergency responses during a maternal cardiac arrest for all providers. Highlights of these guidelines include the following.

**Figure 2:**
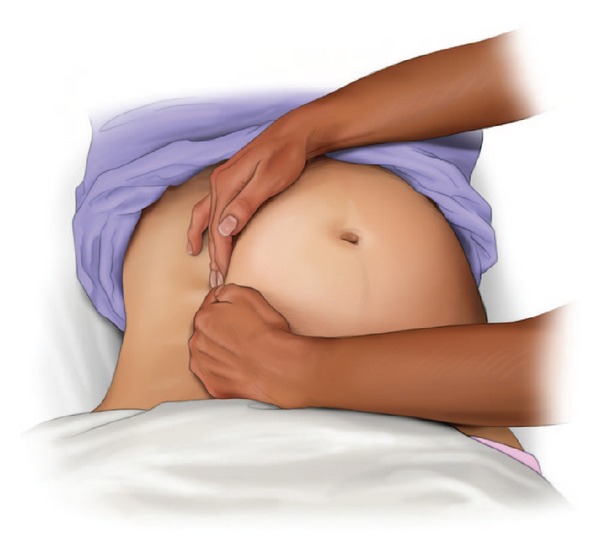
Left uterine displacement with 2-handed technique [3].

**Figure 3:**
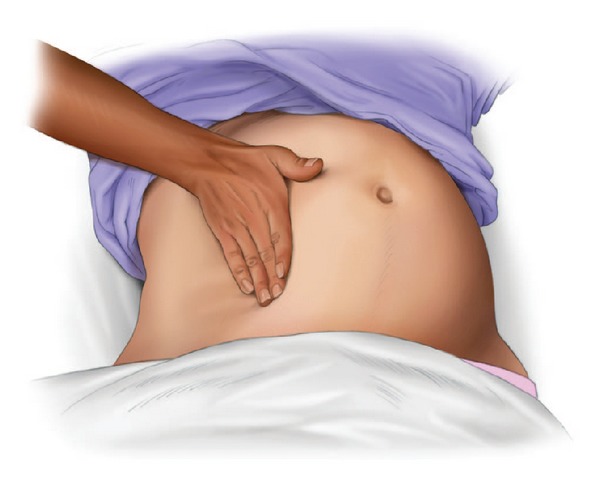
Left uterine displacement using 1-handed technique [3].

1. Coordinate multiple teams during and after the cardiac arrest.
2. Do not delay usual measures such as defibrillation and the administration of medications.
3. Perform aortocaval decompression maneuvers, preferably manual left uterine displacement (LUD) (Figures 2 and 3) [3].
4. Consider the airway difficult, and the most experienced provider should manage the airway.
5. Intravenous access is important but should be placed above the diaphragm.
6. There should be a dedicated timer to document when 4 minutes after the onset of a maternal cardiac arrest have elapsed, in order to make a decision on the need for a PMCS. PMCS should be performed by 5 minutes after the onset of a maternal cardiac arrest if there is no return of spontaneous circulation (ROSC) by 4 minutes with the usual resuscitation measures.
7. Consider an expanded etiology list for the cause of the cardiac arrest; BEAU-CHOPS can be used as a usual mnemonic.

However, in order to understand and properly implement these guidelines, caregivers must have a comprehensive understanding of the unique aspects of maternal resuscitation. Given the lack of science in this area, it is vital that caregivers are educated on the physiological differences during pregnancy and, therefore, the basis for the recent guidelines, algorithm, and previous recommendations.

## 3. The ABCs of Maternal Physiology during Cardiac Arrest

The optimal management of a cardiac arrest in pregnancy must take into account the physiological changes of pregnancy as they relate to resuscitation. There are significant changes in the airway (A), breathing (B), and circulation (C) during pregnancy, and therefore important resuscitation modifications must be made to maximize the chance of a successful resuscitation [3, 16]. 

### 3.1. Airway

Pregnant patients should be viewed as having a difficult airway by all staff involved in maternal resuscitation [3, 19]. Failed intubations even occur in nonarrested pregnant patients undergoing general anesthesia, with an incidence of approximately 1 : 300 [20, 21]. There is ongoing concern related to the incidence of airway-related maternal morbidity and mortality [8], and this has led to the development of specialized maternal airway management strategies and recommendations [19]. During pregnancy, physiological changes in the upper airway include hyperemia, hypersecretion, and edema [22]. These changes increase the friability of the mucosa and may result in impaired visualization and increased bleeding, especially with repeat airway manipulations [23]. In addition, the airway during pregnancy is smaller [24], and, therefore, it is recommended that a smaller endotracheal tube is used during intubation [3]. The topic of optimal airway management as well as protocols for failed intubation and difficult airway management is beyond the scope of this review; however, resources on these specific areas are available [19, 23].

The three important points for emergency staff to understand about airway modifications include the following.

1. Good basic life support can optimize ventilations, chest excursion, and oxygenation and defer the need for an advanced airway.
2. Advanced airway placement is difficult in a maternal cardiac arrest. Thus, it is important to be thoroughly prepared.
3. The most experienced person should secure and manage the advanced airway during a maternal cardiac arrest.

### 3.2. Breathing

During pregnancy there is an increased risk of rapid desaturation [25]. Reduced oxygen reserve is the main physiological reason for the rapid desaturation observed during pregnancy. The reduced oxygen reserve seen during pregnancy is the result of an increase in oxygen consumption [26, 27] coupled with a reduced functional residual capacity [28]. There is also increased intrapulmonary shunting during pregnancy, and, therefore, ventilation-perfusion mismatch will be poorly tolerated in the pregnant patient [29]. Thus, during a cardiac arrest, and especially prior to intubation attempts, oxygenation should be optimized in the pregnant patient [3, 19]; however, resuscitation staff should also be aware of the risk of uterine vasoconstriction and fetal hypoxemia that can occur with maternal respiratory alkalosis as a result of overventilation [30]. 

During pregnancy, the elevated diaphragm may result in the need for lower ventilation volumes. There is concern about the risk of aspiration during maternal cardiac arrest due to the reduced lower esophageal sphincter competency [31–33]. Yet, the routine use of cricoid pressure is no longer recommended in the American Heart Association (AHA) resuscitation guidelines as it may impede laryngoscopy and ventilation and may not prevent aspiration [34, 35]. 

The four main points for emergency staff to understand about breathing modifications include the following.

1. Oxygenate well, monitor, and avoid desaturation.
2. Avoid respiratory alkalosis.
3. Consider adjusting ventilation volumes down.
4. Be aware of the risk of aspiration.

### 3.3. Circulation

**Figure 4:**
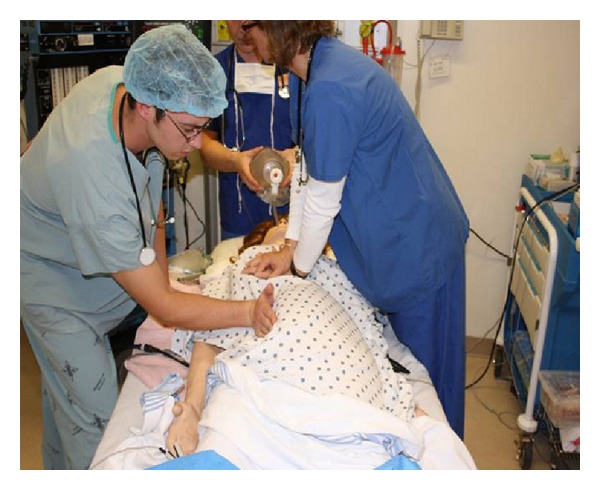
Manual leftward uterine displacement-with resuscitation team [10].

The major circulation concern during a maternal cardiac arrest is the possibility of aortocaval compression caused by the gravid uterus. The gravid uterus can compress the inferior vena cava resulting in a reduced preload and stroke volume [36–38]. We know that by at least 20 weeks gestational age, aortocaval compression is likely to occur [38]; however, even at 12 weeks gestational age, mechanical venous effects of the gravid uterus can be observed [37]. The hemodynamic and cardiovascular effects of uterine compression during cardiac arrest have not been studied. Still, achieving the maximum possible maternal hemodynamic advantage during cardiac arrest and chest compressions is important. Hemodynamic optimization during maternal cardiac arrest in the obviously pregnant patient requires effective aortocaval decompression. The most ideal manner to perform aortocaval decompression is with a manual LUD (see Figures 2, 3, and 4) [3, 10]. Manual LUD allows the patient to remain supine which improves airway access, ease of defibrillation and IV access and enables simultaneous high quality chest compressions. This is important since high quality and effective chest compressions are essential to maximizing the chance of a successful resuscitation in all patients [39–41]. Previously, left lateral tilt had been recommended for aortocaval decompression; however, science has shown that tilt will reduce the forcefulness of the chest compression [13], affecting chest compression quality and potentially negatively impacting survival. 

## 4. Perimortem Cesarean Section

Anoxic brain injury occurs within the 4 minutes after a cardiac arrest is identified. Therefore, if team members are unable to achieve ROSC by 4 minutes in a patient that is obviously gravid, and especially if the patient is >20 weeks gestational age, a decision to perform a PMCS should be made. A PMCS is useful as it allows for complete aortocaval decompression once the uterus is evacuated. There are reports of sudden and dramatic improvements in maternal hemodynamics only after a PMCS [14, 42–45], suggesting that manual maneuvers may not be sufficient for relieving aortocaval compression with the purpose of resuscitation. 

A PMCS should be initiated 4 minutes after the onset of the maternal cardiac arrest, with the aim of delivery by 5 minutes after-onset, if ROSC is not achieved with optimization of the usual resuscitative measures [3]. In order to achieve this goal of delivery within 5 minutes after-onset, the team should prepare for a PMCS as soon as the arrest is documented so that the PMCS incision can be made at the correct time when clinically indicated [3]. The PMCS should be performed at the location where the arrest occurs as transporting the mother to an operating room results in significant delays in delivery time [14, 46]. Furthermore, it is critical that the neonatal team and neonatal resuscitation equipment are on standby to receive the infant once delivered. 

## 5. Etiology and Treatment Considerations

Similar to any cardiac arrest, it is important to consider the etiology of the arrest. There are important differences to highlight in the etiology of maternal deaths and cardiac arrests that emergency department staff should be educated on and aware of when managing a maternal cardiac arrest. Additional etiologies relevant to pregnancy should be considered. BEAU-CHOPS as described in the AHA guideline algorithm on maternal cardiac arrest (Figure 1) can be a useful mnemonic for these additional etiologic considerations which include [3]

1. bleeding/DIC;
2. embolism: coronary/pulmonary/amniotic fluid embolism;
3. anesthetic complications;
4. uterine atony;
5. cardiac disease (MI/ischemia/aortic dissection/cardiomyopathy);
6. hypertension/pre-eclampsia/eclampsia;
7. other: differential diagnosis of standard ACLS;
8. sepsis.

The leading causes of death during pregnancy based on the confidential enquiries into maternal deaths in the United Kingdom, taken from the Centre for Maternal and Child Enquiries (CMACE), include cardiac disease, sepsis, pre-eclampsia/eclampsia, thrombosis/thromboembolism, and amniotic fluid embolism [8]. When a pregnant patient presents in cardiac arrest, these etiologies should be assessed and treated as appropriate. Full recommendations of treatment algorithms for each of these etiologies are outside of the scope of this paper as the topic is immense; however, some important points should be remembered by emergency department staff as follows.

1. Cardiac disease is the most common cause of maternal mortality [8]. The incidence of myocardial infarction [47] and complicated cardiac disease during pregnancy is on the rise based on data from the United States [9]. Fibrinolysis is relatively contraindicated in pregnancy, and, therefore, primary percutaneous coronary intervention is the reperfusion strategy of choice to treat ST segment elevation myocardial infarction in the pregnant woman [3]. Careful consideration as well as discussion between the emergency physician, obstetrician, and cardiologist regarding the pros and cons of different diagnostic and therapeutic strategies is important during acute ischemic events [48, 49]. Nonetheless, postpartum arrest patients with ST-elevated myocardial infarction should be treated urgently with PCI in exactly the same manner as the non-pregnant, postarrest patient.
2. Pregnant women who develop eclampsia may develop a cardiac arrest [8]. Initial management of eclampsia may occur in the emergency department [50] and may include treatment with magnesium sulfate. It should be recognized that magnesium sulfate can cause toxicity and result in cardiac arrest [51]. If a pregnant woman is receiving magnesium sulfate and has a cardiac arrest, empiric treatment with calcium and discontinuing magnesium treatment is advised (Figure 1) [3].
3. Life-threatening amniotic fluid embolisms have been reported and are one of the leading causes of maternal mortality [8, 52]. Treatments with emergency perimortem cesarean section [52] and emergency coronary bypass [53] have been successful.

## 6. Emergency Preparedness

 Maternal cardiac arrest is the most complicated arrest scenario. There are two patients, the mother and the fetus, and multiple teams who normally do not work together are required to have exceptional teamwork skills in order to achieve the best possible outcome. Yet, since maternal arrest is such a rare event, preparations for maternal cardiac arrests are minimal and receive the least attention. This means that when faced with a maternal cardiac arrest, it can be very stressful for all providers as they are dealing with an extremely complex situation that places a high degree of psychological stress on all team members. Therefore, careful and proactive emergency planning should be undertaken at all institutions and across all teams that should be involved in the comprehensive care of a maternal cardiac arrest patient. A multidisciplinary approach to emergency preparedness that can be used by institutions as a guide for emergency preparedness has been previously published in the obstetrical literature [2]. Highlights of this paper include a 10-step approach to the important aspects of emergency preparedness for a maternal cardiac arrest [2]. 

### 6.1. The Team

 The maternal cardiac arrest team should be comprised of the adult resuscitation team, obstetrical team (obstetrician and obstetrical nurse), anesthesia team (anesthesiologist and anesthesiology assistant), and the neonatology team (neonatologist and neonatal nurse and neonatal respiratory therapist) [2]. 

### 6.2. The Equipment

 The suggested equipment includes anything necessary for a perimortem cesarean section and neonatal resuscitation. Suggested components are listed in Table 1 [2].

### 6.3. Implementation

 An important point to consider when implementing the 2010 AHA guidelines for maternal resuscitation is ensuring that there is a specific method of gathering all team members and necessary equipment to the maternal arrest in a timely manner [2]. Training as well as coordination between the involved team members is essential. The worst outcomes for maternal arrest are for out-of-hospital arrest [1], so institutional preparedness should involve coordination with local emergency medical services (EMS). The EMS staff should alert ED staff of any unstable pregnant patient or maternal cardiac arrest patient before their arrival to allow sufficient time to call the maternal code team and ensure they are in the ED upon arrival. 

## 7. Postarrest Care

 The post-arrest (and prearrest/unstable) pregnant patient should be placed at 90° left lateral tilt to relieve possible aortocaval compression. The use of therapeutic hypothermia when appropriate for the non-pregnant patient should be considered in pregnancy [3]. The use of therapeutic hypothermia during pregnancy is a relative contraindication [54]. There have been reports of its successful use in pregnancy; however, one case reported fetal demise, but the patient also had other reasons for a poor outcome, including a prolonged arrest time [55–57]. The use of therapeutic hypothermia should be considered on a case by case basis as maternal cardiac arrest patients were excluded from all prior trials [58, 59]. If therapeutic hypothermia is not applied, all maternal cardiac arrest patients should be well monitored post arrest to ensure normothermia and avoid any hyperthermia. The overarching concern with the use of therapeutic hypothermia in the bleeding or post-PMCS patient relates to the theoretical increased risk of impairing coagulation. Patients receiving therapeutic hypothermia should be monitored for fetal bradycardia. 

## 8. Conclusion

The management of maternal cardiac arrest is very complicated. This review has included the most up-to-date science and guidelines published in the field of maternal resuscitation based on unique and important physiological differences, etiology, and implementation factors that need to be considered in the advance planning and delivery of an optimal emergency response to a maternal cardiac arrest. 

### 8.1. Permissions

Permission has been provided for use of all figures and tables included in this manuscript. Permission to reprint Table 1 has been provided courtesy of the Society of Obstetricians and Gynaecologists of Canada. Permission to reprint Figure 4 has been provided courtesy of Elsevier. 

## Figures and Tables

**Table 1:** Recommended PMCS and neonatal equipment [2].

Equipment contents of the emergency caesarean section tray	Equipment for neonatal resuscitation and stabilization
Scalpel with no. 10 blade	Overbed warmer
Lower end of Balfour retractor	Neonatal airway supplies
Pack of sponges	Umbilical access
2 Kelly clamps	Medications (e.g., epinephrine 1 : 10 000)
Needle driver	
Russian forceps	
Sutures and suture scissors	
